# The efficacy and safety of Rhodiola formulation for the treatment of ischemic heart disease: A protocol for systematic review and meta-analysis

**DOI:** 10.1097/MD.0000000000031736

**Published:** 2022-11-11

**Authors:** Shengguo Teng, Xu Qian, Jianghong Zheng, Jun Qian

**Affiliations:** a Department of Integrated Chinese and Western Medicine, Zhangye People’s Hospital affiliated to Hexi University, Gansu, China; b Department of Traditional Chinese Medicine, Zhangye People’s Hospital affiliated to Hexi University, Gansu, China; c Department of Cardiovascular Medicine, Zhangye People’s Hospital affiliated to Hexi University, Gansu, China; d Department of outpatient, Zhangye People’s Hospital affiliated to Hexi University, Gansu, China.

**Keywords:** ischemic heart disease, meta-analysis, protocol, Rhodiola formulation, systematic review

## Abstract

Ischemic heart disease is the main cause of heart failure, which seriously endangers the health of people and puts a huge burden on health care resources all over the world. We propose the current protocol to evaluate the effectiveness and safety of Rhodiola on ischemic heart disease, providing a reference for clinical use.

**Methods:**

Two research members will electronically and independently search 4 English databases (EMBASE, PubMed, National Guideline Clearinghouse, and Cochrane Central Register of Controlled Trials) and 4 Chinese databases (Chinese Biomedical Literature Database, Chinese National Knowledge Infrastructure, Wanfang Database, and VIP Database) from their inception to October 2020. Quality assessment of the included randomized controlled trial was assessed using the Cochrane Collaboration’s tool. All calculations were carried out with Stata 11.0 (The Cochrane Collaboration, Oxford, United Kingdom).

**Results:**

A synthesis of current evidence of Rhodiola formulation for ischemic heart disease will be provided in this protocol.

**Conclusion:**

This study will provide a theoretical basis for the clinical use of Rhodiola formulation for treating ischemic heart disease.

## 1. Introduction

Cardiovascular disease has been a serious threat to human life for a long time and its incidence is increasing.^[1,2]^ Ischemic heart disease (IHD), also known as coronary artery disease, is the main cause of heart failure, which seriously endangers the health of people and puts a huge burden on health care resources all over the world.^[3,4]^ In China, more than half of patients with heart failure developed from IHD.^[5]^ Therefore, it is a common problem for clinicians and researchers to explore the pathogenesis of IHD and carry out effective prevention and treatment. According to clinical studies, the onset of IHD is a long-term and complex process, which mainly involves pathophysiological changes such as coronary artery stenosis, coronary artery thrombosis and embolism, vasospasm, microcirculation disorder, inflammation, endothelial dysfunction, apoptosis, and autophagy cascading reactive activation, etc.^[6–8]^

Currently, the main treatment for IHD mainly includes drug therapy, percutaneous coronary intervention, and coronary artery bypass grafting.^[9,10]^ However, it could result in certain side effects and poor compliance. Traditional Chinese medicine is an important part of complementary and alternative medicine, which has been widely accepted in China and applied in practice. Rhodiola (Crassulaceae) is a famous genus of medicinal herbs in China. In traditional Chinese medicine, Rhodiola species have been used as a tonic, hemostatic, or antibechic agent for treatment of leucorrhea, contusion, cold/flu-like symptoms and maintain healthy cardiovascular function.^[11–13]^ Phytochemical studies have revealed that the main bioactive compounds, salidroside and tyrosol are similar to those used in modern medicine for improving cardiovascular function. These compounds exert pharmacological effects, such as enhancing myocardial contractility, increasing myocardial contraction and inducing hypotension in laboratory animals.^[14]^

However, to our knowledge, there is no systematic review of its efficacy and safety in the treatment of IHD. Therefore, we propose the current protocol to evaluate the effectiveness and safety of Rhodiola on IHD, providing a reference for clinical use.

## 2. Methods

### 2.1. Study registration

The protocol of this review was registered in PROSPERO (CRD42022368148). Meanwhile, it was reported as per the statement guidelines of preferred reporting items for the systematic review and meta-analysis protocol.^[15]^ Given that the meta-analysis is a secondary research which based on some previously published data, ethical approval is not necessary for our research.

### 2.2. Electronic searches

Two research members will electronically and independently search 4 English databases (EMBASE, PubMed, National Guideline Clearinghouse, and Cochrane Central Register of Controlled Trials) and 4 Chinese databases (Chinese Biomedical Literature Database, Chinese National Knowledge Infrastructure, Wanfang Database, and VIP Database) from their inception to October 2020. The searched items will be used as follows: Rhodiola, ischemic heart disease, and randomized controlled trial. The same terms will be searched in the Chinese databases. The data will be retrieved with the combination of medical keywords and uncontrolled terms. The detailed retrieval strategy of PubMed database will be shown in the supplemental digital content (Table 1), and will be constantly modified by searching other databases.

**Table 1:** Search strategy for PubMed.

#1 Rhodiola formulation[Title/Abstract]
#2 Rhodiola rosea[Title/Abstract]
#3 Rhodiola sacra[Title/Abstract]
#4 Rhodiosin[Title/Abstract]
#5 Rhodioloside[Title/Abstract]
#6 Root of Kirilow Rhodiola[Title/Abstract]
#7 Roseroot[Title/Abstract]
#8 Chinese herbal medicine[Title/Abstract]
#9 Traditional Chinese medicine[Title/Abstract]
#10 #1 OR #2 OR #3 OR #4 OR #5 OR #6 OR #7 OR#8 OR#9
#11 Ischemic heart disease[Title/Abstract]
#12 Coronary artery disease[Title/Abstract]
#13 Myocardial ischemia[Title/Abstract]
#14 Coronary atherosclerotic heart disease[Title/Abstract]
#15 #11 OR #12 OR #13 OR #14
#16 #10 AND #15

### 2.3. Inclusion and exclusion criteria

Inclusion criteria: Only randomized controlled trials in English or Chinese were included regardless of being published or unpublished; Rhodiola formulation medicine as a sole agent or in combination with other drug regimens compared to other Chinese formulations or other drugs for treating IHD; the formulation had to be produced by a pharmaceutical factory and in accordance with the guidelines of World Health Organization; the diagnoses of IHD were based on standardized diagnostic criteria, such as Nomenclature and Criteria for Diagnosis of Ischemic Heart Disease/World Health Organization or other diagnostic criteria for IHD. Exclusion criteria: the study involved only animal studies or in vitro studies; review, comment and letter; the report represented duplicate publications of other studies; the dosage of the Rhodiola formulation was not clearly reported.

### 2.4. Study selection

**Figure 1.**
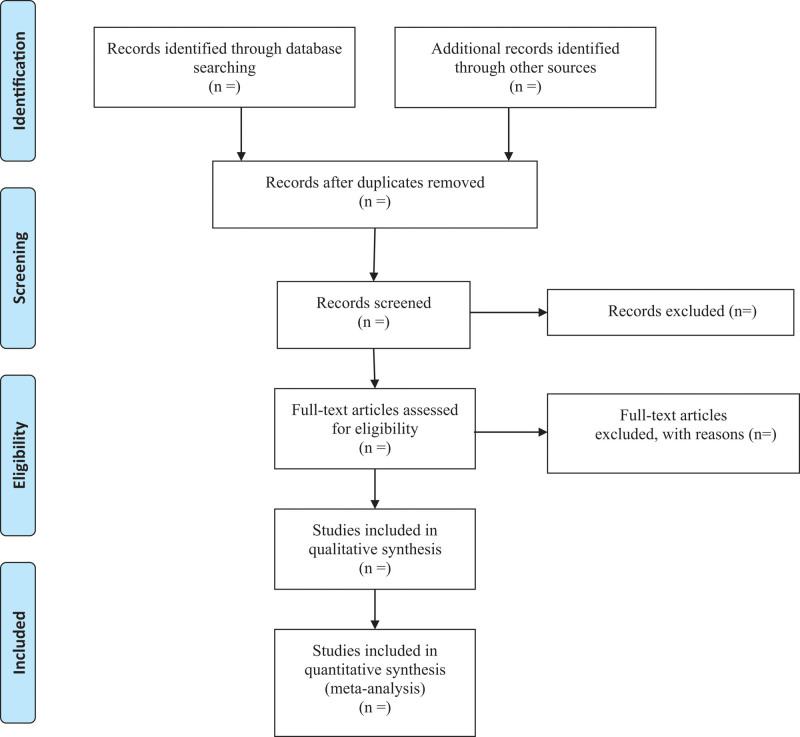
Flow diagram of study selection process.

First of all, all qualified documents will be extracted in the form of title and abstract, and preliminary screening will be conducted based on this information. On the basis of the previous step, the full text of the qualified literature will be obtained and further screened. All screening processes will be performed independently by the 2 authors, and the reasons for each rejection will be documented. Disagreements between the 2 reviewers will be resolved by discussing with the third investigator. A PRISMA-compliant flow chart (Fig. 1) will be used to describe the selection process of eligible literatures.

### 2.5. Data extraction

A standard form for date extraction is printed for date extraction. Two authors independently extracted the relevant data from the included articles. Details of incomplete data of included studies are obtained by consulting the corresponding author. Following data was extracted: First author names, published year, sample size, study design, comparable baseline, dosage of Rhodiola formulation, and duration of follow-up. Other relevant data was also extracted from individual studies.

### 2.6. Risk of bias

Quality assessment of the included randomized controlled trial was assessed by 2 authors independently which used the Cochrane Collaboration’s tool.^[16]^ We conducted “risk of bias” table including the following key points: random sequence generation, allocation concealment, blinding, incomplete outcome data, free of selective reporting and other bias, each item was recorded by “Yes,” “No,” or “Unclear.”

### 2.7. Statistical methods

All calculations were carried out with Stata 11.0 (The Cochrane Collaboration, Oxford, United Kingdom). Statistical heterogeneity was assessed based on the value of *P* and *I*^2^ using the standard chi-square test. When *I*^2^ > 50%, *P* < .1 was considered to be significant heterogeneous. The random-effect model was performed for meta-analysis; otherwise, the fixed-effect model was used. When possible, subgroup analyses were conducted to explore the origins of the heterogeneity. The results of dichotomous outcomes were expressed as risk difference (RD) with a 95% confidence intervals (CIs). For continuous various outcomes, mean difference (MD) or standard MD with a 95% CIs was applied. Subgroup analysis was conducted according to the dosage of Rhodiola formulation.

## 3. Discussion

IHD is a dynamic process of atherosclerosis of the coronary arteries or functional alterations of coronary circulation that can be modified by lifestyle, pharmacological therapies, and revascularization.^[17]^ It still represents a large burden on individuals and health care resources worldwide. Rhodiola formulations can promote blood flow, enhance myocardial contractility and reduce mean arterial pressure. Furthermore, Rhodiola extracts have been shown to have anti-arrhythmic activity, protective effects in ischemia/reperfusion (IR) and hypoxia-induced cardiomyocyte cell death and prevent IR-induced ventricular tachycardia,^[18,19]^ all of which are plausible mechanisms for treating IHD. This article provides a PRISMA-compliant systematic review and meta-analysis on the efficacy of Rhodiola in the treatment of IHD. To our knowledge, no other similar review has been published. We hope that this paper can provide strong evidence for the clinical use of Rhodiola formulation for the treatment of IHD.

## Author contributions

**Conceptualization:** Xu Qian.

**Methodology:** Jianghong Zheng.

**Writing – original draft:** Shengguo Teng.

**Writing – review & editing:** Jun Qian.
